# Effects of internal and external attentional focus on dart-throwing performance: a systematic review

**DOI:** 10.3389/fpsyg.2026.1923857

**Published:** 2026-09-03

**Authors:** Ning Feng, Qiongqi Shangguan, Xuanyong Zhu

**Affiliations:** Department of Sport Science, College of Natural Science, Jeonbuk National University, Jeonju, Republic of Korea

**Keywords:** attentional focus, dart throwing, external focus of attention, internal focus of attention, motor learning, motor performance

## Abstract

**Background:**

Attentional focus is a central issue in motor control and motor learning research. Existing evidence generally suggests that an external focus of attention (EF) is more beneficial than an internal focus of attention (IF); however, findings in the context of dart throwing remain inconsistent and may be influenced by factors such as task demands, test phase, and learner characteristics. This systematic review aimed to synthesize the effects of EF and IF on dart-throwing performance and to examine potential sources of variation in these effects.

**Methods:**

A systematic search was conducted in PubMed, Web of Science, Scopus, and Embase, covering all records from database inception to February 27, 2026. Experimental studies comparing the effects of EF and IF on dart-throwing performance in healthy individuals were included. Methodological quality was assessed using the mixed methods appraisal tool (MMAT). Given variation in study designs, outcome reporting, and assessment phases, together with insufficient information for deriving comparable effect estimates in some studies, the evidence was synthesized narratively.

**Results:**

A total of 19 studies comprising 23 experimental units and 923 participants were included. During acquisition, EF-favoring effects were observed in several studies, but the overall findings were mixed, with conditional and non-significant results also frequently reported. Some retention studies also favored EF, whereas transfer evidence remained sparse and inconsistent. Overall, attentional focus effects in dart throwing appear to be context dependent, with their direction and magnitude potentially varying across testing phases, task demands, practice and test arrangements, and learner characteristics.

**Conclusions:**

The evidence does not support a uniform advantage of either attentional focus strategy in dart throwing. Rather, EF–IF effects appear to be context dependent and sensitive to the phase of testing and study conditions. These findings support a more task-specific and conditional interpretation of attentional focus effects, highlighting the need for standardized research with delayed retention and transfer assessments before firm instructional recommendations can be made.

**Systematic review registration:**

https://www.crd.york.ac.uk/PROSPERO/view/CRD420261363437, identifier PROSPERO, CRD420261363437.

## 1 Introduction

Attentional focus is a central topic in motor control and motor learning research and is typically categorized as either internal focus of attention or external focus of attention. An internal focus directs attention to one's own bodily movements or movement processes, whereas an external focus directs attention to the external effects of the movement or its environmental outcomes (Wulf et al., 1998; Wulf, 2013). A substantial body of research has suggested that, across a range of motor tasks, an external focus is often more beneficial than an internal focus and may facilitate both movement execution and skill acquisition (Wulf and Prinz, 2001; Nicklas et al., 2024; Chua et al., 2021). However, some studies have also noted that the apparent advantage of an external focus may be influenced by publication bias and between-study heterogeneity, and that its effects may vary across specific contexts (McKay et al., 2024). Accordingly, the advantage of an external focus may be more appropriately understood as a general tendency rather than a universal principle that holds consistently across all tasks and situations.

Among the proposed explanations for the advantage of EF, the constrained action hypothesis has been the most influential. This hypothesis posits that IF is more likely to induce conscious control over movement processes, thereby disrupting the relatively automatic regulation of action. By contrast, EF is thought to facilitate the natural organization and efficient execution of the motor system (Wulf et al., 2001). OPTIMAL theory further proposes that EF enhances motor performance and learning by strengthening goal-action coupling, reducing self-focus, and interacting with motivational factors (Wulf and Lewthwaite, 2016). Accordingly, the effects of attentional focus may be reflected not only in differences in behavioral outcomes, but also in changes in movement control and execution efficiency.

Dart throwing is a goal-directed throwing task that has frequently been used to manipulate attentional focus through explicit instructional cues and to examine their effects on throwing performance. In comparisons between IF and EF, the dart-throwing task allows researchers to operationally distinguish attentional orientations through explicit instructions directing attention toward bodily movements or movement processes, vs. movement effects or target-related outcomes (Wulf et al., 2010). It has therefore become one of the more representative experimental tasks in this line of research. However, findings from the existing dart-throwing literature are not entirely consistent. Some studies have shown that EF can improve throwing accuracy and may be accompanied by lower electromyographic activity and more favorable movement characteristics (Lohse et al., 2010; Hitchcock and Sherwood, 2018). Other studies, however, have reported either no significant EF–IF difference or only a trend favoring EF, suggesting that inconsistent findings may be related to factors such as skill level, instructional cues, and additional task constraints (Schorer et al., 2012; Neugebauer et al., 2020; Asadi et al., 2022). At the same time, existing studies still differ in experimental design, outcome measures, and the timing of testing, which makes direct comparison and integrated interpretation across studies more difficult.

Although a growing number of systematic reviews and meta-analyses have examined attentional focus, most have approached the topic from a cross-task or cross-sport perspective and have focused on its overall effects (Zang et al., 2025; Starzak et al., 2024; Nicklas et al., 2024). In contrast, a systematic synthesis centered specifically on dart throwing remains lacking. As a classic experimental task in attentional focus research, dart throwing has accumulated a meaningful body of empirical evidence; however, the findings are not entirely consistent. Compared with broad discussions of attentional focus effects across different tasks, a task-specific focus on dart throwing may allow for a more precise understanding of the direction, consistency, and potential sources of variation in the available evidence. Therefore, the present study aimed to systematically review experimental research on the effects of attentional focus on dart-throwing performance and to further examine potential sources of variation related to study methodology, task and practice conditions, and learner characteristics, thereby providing more specific evidence to inform future research design and practical application.

## 2 Materials and methods

### 2.1 Protocol and registration

This systematic review was conducted in accordance with the Preferred Reporting Items for systematic reviews and meta-analyses (PRISMA) guidelines (Page et al., 2021) and was registered in the International Prospective Register of Systematic Reviews (PROSPERO) under registration number CRD420261363437.

### 2.2 Search strategy

A systematic literature search was conducted in PubMed, Web of Science, Scopus, and Embase. The final database search was conducted on February 27, 2026, covering all records from database inception to that date. The search strategy was developed around two core concepts, “dart throwing” and “attentional focus,” and was adapted to the search rules of each database with respect to keywords, field tags, and proximity operators (Supplementary Table 1). In addition to the database search, the reference lists of included studies were manually screened, and citation tracking was used to identify additional relevant studies.

### 2.3 Study selection

All retrieved records were first imported into EndNote 2025 for duplicate removal. After deduplication, two reviewers independently screened the titles and abstracts to exclude studies that clearly did not meet the eligibility criteria. The full texts of potentially eligible studies were then obtained and assessed independently against the predefined inclusion and exclusion criteria. Any disagreements between the two reviewers were resolved through discussion; if consensus could not be reached, a third reviewer made the final decision. The study selection process and reasons for exclusion were presented in a PRISMA 2020 flow diagram.

### 2.4 Eligibility criteria

The inclusion and exclusion criteria were established according to the PICOS framework (Amir-Behghadami and Janati, 2020). The inclusion criteria were as follows: (1) Participants: healthy individuals without neurological disorders, developmental disorders, psychiatric conditions, or other impairments that could affect motor control or motor learning; no restriction was placed on skill level. (2) Intervention: studies that directly manipulated attentional focus through explicit IF and EF instructions, delivered orally or in writing, during a dart-throwing task. (3) Comparison: studies were required to include both an IF condition and an EF condition for direct comparison. (4) Outcomes: studies had to report at least one quantifiable dart-throwing performance outcome, such as accuracy, error, or score. (5) Study design: experimental studies, including randomized controlled trials, non-randomized experimental studies, and within-subject experimental studies in which condition order was randomized or counterbalanced.

The exclusion criteria were as follows: (1) studies involving non-healthy participants, including but not limited to individuals with neurological disorders, developmental disorders, severe psychiatric conditions, or other diseases or functional impairments that might influence motor control or motor learning performance; (2) studies in which attentional focus was manipulated only indirectly through secondary judgment, estimation, or reporting tasks, without explicit IF and EF instructions; (3) studies that did not include a comparison between IF and EF; (4) studies that did not report quantitative dart-throwing performance outcomes; and (5) reviews, conference abstracts, studies for which the full text was unavailable, or records that did not address the research question of the present review.

### 2.5 Data extraction

After duplicate removal and preliminary screening in EndNote 2025, relevant data were extracted from the eligible studies using a predesigned Excel 2021 data extraction form. Data extraction was performed independently by two reviewers, and any disagreements were resolved by a third reviewer. The extracted information primarily included the author(s) and year of publication, participant characteristics, study design, attentional focus instructions, instruction mode, practice dosage, manipulation checks, outcome measures, and assessment time points. For articles reporting multiple distinct experiments, each experiment was treated as a separate experimental unit for descriptive synthesis, whereas each article was counted only once when reporting the total number of included studies. Within each experimental unit, multiple conditions were extracted when they were clearly defined and directly relevant to the research question, while preserving the overall study design and condition structure. In addition, to ensure consistent classification of participant skill level across studies, the original classification was retained when a study explicitly identified participants as novices. When novice status was not explicitly stated but the frequency of prior dart-throwing participation was reported, participants who had engaged in dart throwing three times or fewer in the previous year were classified as novices.

### 2.6 Methodological quality assessment

The methodological quality of the included studies was assessed using the mixed methods appraisal tool (MMAT). The MMAT is a methodological appraisal tool applicable to different study designs (Hong et al., 2018) and enables a systematic evaluation of the appropriateness of study design, data collection, and analytical methods according to study type (Pace et al., 2012). For the experimental studies included in this review, those involving between-group random allocation or within-subject randomization or counterbalancing of condition order were assessed using the relevant MMAT category based on their primary allocation approach and original design characteristics. Specifically, studies with clear random allocation features were classified as quantitative randomized controlled trials, whereas studies without explicit random allocation but with experimental manipulation were classified as quantitative non-randomized studies. Methodological quality was assessed at the study level. For studies containing multiple experimental units, the appraisal was based on the overall original study design rather than on each experimental unit separately. Quality assessment was conducted independently by two reviewers. Any disagreements were resolved through discussion, and, when necessary, a third reviewer was consulted. In accordance with MMAT guidance, no overall score was calculated for each study; instead, the methodological quality of the included studies was described on the basis of the results for each appraisal criterion (Hong et al., 2019).

### 2.7 Data synthesis

Given differences in study designs, outcome reporting, and assessment phases, together with insufficient information for deriving comparable effect estimates in some studies, the findings were synthesized narratively. This approach was guided by the SWiM reporting guideline and recommendations for narrative synthesis (Campbell et al., 2020; Popay et al., 2006). Direct-comparison findings were grouped by test phase and categorized as significant EF-favoring findings, conditional or mixed findings, or no significant EF–IF difference. Instructional and practice characteristics were additionally summarized descriptively to explore potential methodological variation across studies.

## 3 Results

### 3.1 Study selection

**Figure 1:**
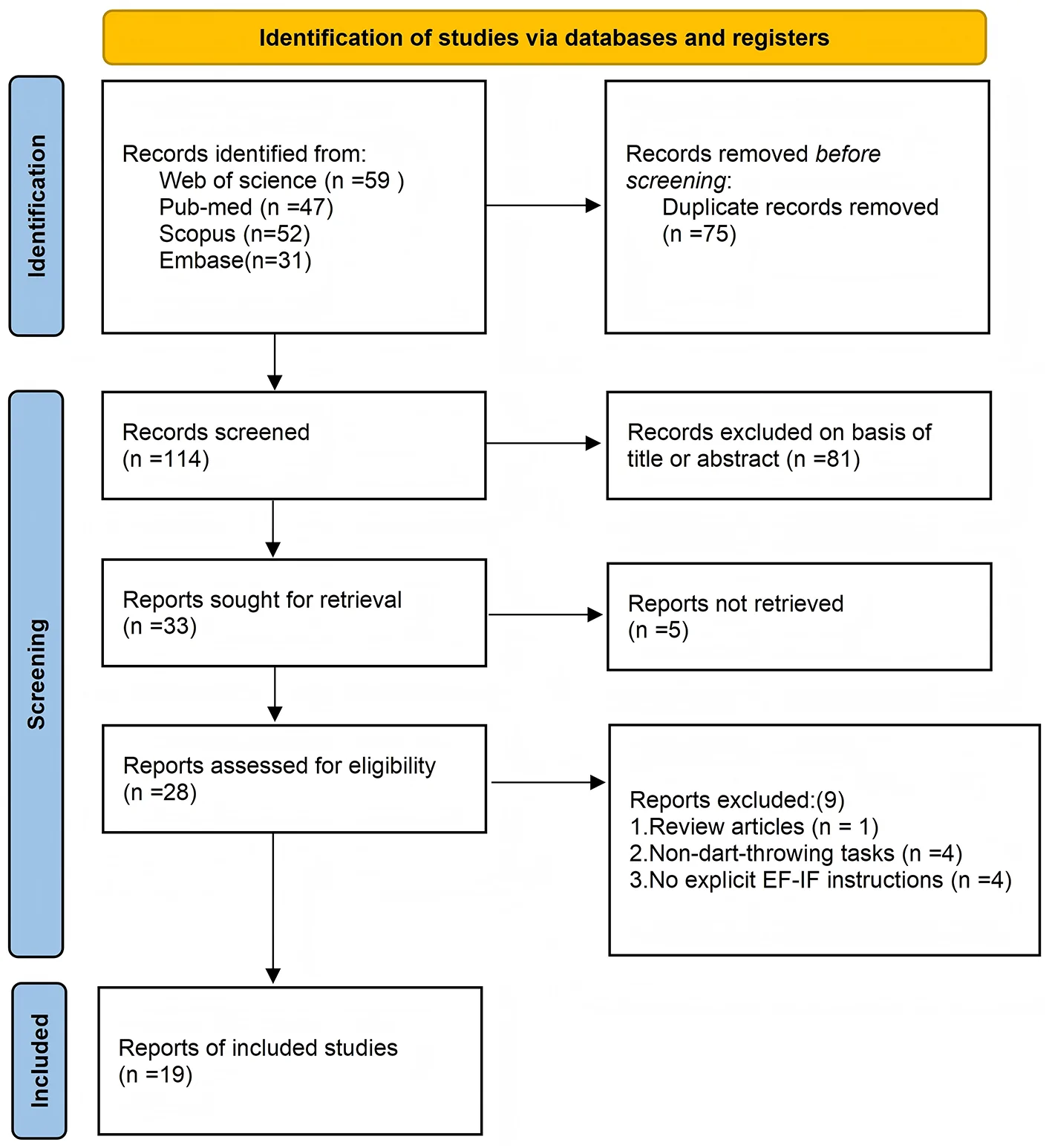
PRISMA 2020 flow diagram of study selection. Adapted from Page et al. (2021). License: CC BY 4.0. Source: PRISMA 2020 Flow Diagram (https://www.prisma-statement.org/prisma-2020-flow-diagram).

The initial database search identified 189 records. After 75 duplicate records were removed, 114 records were screened by title and abstract, and 81 records were excluded. A total of 33 reports were sought for retrieval, of which 5 could not be retrieved. Therefore, 28 full-text articles were assessed for eligibility. Of these, 1 was a review article, 4 did not involve a dart-throwing task, and 4 used indirect attentional manipulations rather than explicit and directly comparable IF and EF instructions. Ultimately, 19 studies were included in the review (Figure 1).

### 3.2 Study characteristics

A total of 19 studies were included in this review, comprising 23 experimental units and a combined sample of 923 participants. Most participants were novices, with only a small number of experimental units including experienced performers or experts. Participants were predominantly young adults, although a small number of studies also included children. Across the 23 experimental units, 7 used within-subject designs, 7 used between-subject designs, and 9 used mixed designs. The included studies primarily focused on comparisons between EF and IF. A few studies adopted more continuous or specialized attentional focus manipulations, and some also incorporated additional factors related to task context, practice conditions, and individual characteristics. All outcome measures were related to dart-throwing accuracy, with mean radial error being the most commonly reported measure. Other outcomes included absolute error, radial distance, scores, and error scores. In addition to acquisition-phase assessments, some studies also included retention and transfer tests (Table 1).

**Table 1:** General characteristics of the included experimental units.

Study	*n*	Age	Skill level	Design	Additional conditions	Outcome measure	Test stage
Asadi et al. (2022)	18	23.89 ± 3.79	Novice	Within-subject	Low vs. high cognitive load	Mean radial error	AQ
Lohse et al. (2014a)	15	Not reported	Novice	Within-subject	Free focus condition; multiple focus distances	Absolute error	AQ
Ghorbani et al. (2019)	36	21.16 ± 1.85	Novice	Between-subject	None	Scores	AQ, RT, TT
Hitchcock and Sherwood (2018)	22	20.2 ± 3.28	Novice/Experienced	Within-subject	None	Mean radial error	AQ
Becker and Fairbrother (2019)	22	Not reported	Novice	Between-subject	None	Error scores	AQ, RT
Lohse et al. (2010)	12	Not reported	Not reported	Within-subject	None	Absolute error	AQ
Neugebauer et al. (2020)	36	Not reported	Novice	Between-subject	Visual vs. kinesthetic focus	Radial distance	AQ
Querfurth et al. (2016)	20	25.8 ± 3.9	Novice	Within-subject	None	Mean radial error	AQ
Sampson et al. (2025)	36	20.65 ± 1.94	Not reported	Within-subject	Motivational self-talk	Mean radial error	AQ
Schorer et al. (2012)	12	Expert: 33.2 ± 4.2; Novice: 27.6 ± 2.6	Expert/Novice	Mixed design	KR vs. no-KR; skill level	Mean radial error	AQ
Sherwood et al. (2020), Exp. 1/2/3/4	56	18.6 ± 0.94	Novice/Experienced	Mixed design	Tone-estimation dual task	Mean radial error	AQ
	54	19.5 ± 1.12	Novice/Experienced	Mixed design	Tone-estimation dual task	Mean radial error	AQ
	72	19.3 ± 1.94	Not reported	Mixed design	Dart-goal vs. force-goal task	Mean radial error	AQ
	48	20.1 ± 1.7	Novice/Experienced	Mixed design	Force level (20% vs. 50% MF); dart-goal vs. force-goal task	Mean radial error	AQ
Simpson et al. (2022)	36	33.6 ± 19.7	Novice	Between-subject	None	Mean radial error	AQ, RT, TT
Tse and van Ginneken (2017)	60	10.4 ± 1.8	Novice	Between-subject	High vs. low conscious control propensity	Mean radial error	AQ, RT, TT
van Ginneken et al. (2017)	46	21.3 ± 1.8	Novice	Mixed design	Conscious monitoring vs. conscious control; order; CMP/MS-C	Mean radial error	AQ
Marchant et al. (2009)	72	19.82 ± 3.78	Novice	Within-subject	Sequence effect; preference effect	Error scores	AQ
Emanuel et al. (2008)	66	Children: 9.04 ± 0.35 Adults: 28.73 ± 4.23	Novice	Mixed design	None	Mean radial error	AQ, RT, TT
Kakvandi et al. (2025)	48	9.67 ± 0.72	Novice	Mixed design	High vs. low visuospatial WMC; longer-distance transfer; dual-task transfer	Mean radial error	AQ, RT, TT1/2
Lohse et al. (2014b), Exp. 1/2	40	Not reported	Novice	Mixed design	Acquisition focus × testing focus; short acquisition; wrist-weight transfer	Mean radial error	AQ, RT, TT
	48	Not reported	Novice	Mixed design	Acquisition focus × testing focus; longer acquisition; wrist-weight transfer	Mean radial error	AQ, RT, TT
Shafizadeh et al. (2013)	48	22.5 ± 3.6	Novice	Mixed design	Physical vs. observational practice; self-efficacy controlled	Scores	AQ, RT, TT

EF, external focus; IF, internal focus; FOA, focus of attention; AQ, acquisition phase; RT, retention test; TT, transfer test; KR, knowledge of results; no-KR, no knowledge of results; CMP, conscious motor processing; MS-C, movement self-consciousness; MF, maximal force; WMC, working memory capacity.

Additional descriptive examination revealed considerable variation in attentional focus instructions and practice protocols across studies (Supplementary Table 2). IF cues targeted different components of the throwing movement, whereas EF cues varied in their emphasis on the dart, its flight or trajectory, and target-related outcomes. Practice dosage also differed substantially in the number of throws per block, the number of blocks, and the overall amount of practice. However, no consistent descriptive pattern was apparent between either instruction content or practice dosage and the direction of EF–IF findings.

### 3.3 Methodological quality

The MMAT appraisal showed variation in methodological quality across the included studies. Based on their primary allocation approach and original design characteristics, 10 studies were classified as quantitative non-randomized studies and nine as quantitative randomized controlled trials. Among the non-randomized studies, outcome and intervention measurements were generally appropriate, although uncertainty remained regarding sample representativeness, completeness of outcome data, and intervention implementation in some studies. Among the randomized controlled trials, outcome data were generally complete, but randomization procedures and blinding of outcome assessors were frequently insufficiently reported. Overall, the appraisal identified recurring limitations primarily in the reporting of randomization, blinding, and selected study procedures (Table 2).

**Table 2:** Methodological quality appraisal of the included studies using the MMAT.

Study type	Study	Item 1	Item 2	Item 3	Item 4	Item 5
Quantitative non-randomized	Asadi et al., 2022	Yes	Yes	Yes	Yes	Yes
	Lohse et al., 2014a	Yes	Yes	Yes	Yes	Yes
	Hitchcock and Sherwood, 2018	Yes	Yes	Yes	Yes	Yes
	Lohse et al., 2010	Can't tell	Yes	Can't tell	Yes	Yes
	Querfurth et al., 2016	Yes	Yes	Can't tell	Yes	Can't tell
	Sampson et al., 2025	Yes	Yes	Yes	Yes	Yes
	Schorer et al., 2012	Can't tell	Yes	Can't tell	Can't tell	Can't tell
	Sherwood et al., 2020	Can't tell	Yes	Yes	Yes	Can't tell
	Lohse et al., 2014b	Can't tell	Yes	Yes	Can't tell	Yes
	Shafizadeh et al., 2013	Can't tell	Yes	Yes	Yes	Yes
Quantitative randomized controlled trial	Ghorbani et al., 2019	Can't tell	Yes	Yes	Can't tell	Yes
	Becker and Fairbrother, 2019	Can't tell	Yes	Yes	Can't tell	Yes
	Neugebauer et al., 2020	Can't tell	No	Can't tell	Can't tell	Can't tell
	Tse and van Ginneken, 2017	Can't tell	Yes	Yes	Can't tell	Yes
	van Ginneken et al., 2017	Can't tell	Can't tell	Yes	Can't tell	Can't tell
	Simpson et al., 2022	Can't tell	Yes	Yes	Can't tell	Yes
	Marchant et al., 2009	Can't tell	Can't tell	Can't tell	Can't tell	Yes
	Emanuel et al., 2008	Can't tell	Can't tell	Yes	Can't tell	No
	Kakvandi et al., 2025	Can't tell	Yes	Yes	Can't tell	Can't tell

Study type indicates the MMAT category applied to each study. For quantitative non-randomized studies, Item 1 = Are the participants representative of the target population? Item 2 = Are measurements appropriate regarding both the outcome and intervention (or exposure)? Item 3 = Are there complete outcome data? Item 4 = Are the confounders accounted for in the design and analysis? Item 5 = During the study period, is the intervention administered (or exposure occurred) as intended? For randomized controlled trials, Item 1 = Is randomization appropriately performed? Item 2 = Are the groups comparable at baseline? Item 3 = Are there complete outcome data? Item 4 = Are outcome assessors blinded to the intervention provided? Item 5 = Did the participants adhere to the assigned intervention?

### 3.4 Direct comparisons of the effects of EF and IF on dart-throwing performance

#### 3.4.1 Acquisition phase

A total of 10 direct-comparison studies reported the effects of EF and IF on dart-throwing performance during the acquisition phase. The findings were mixed. Three studies provided statistically significant evidence favoring EF, reflected either in lower error under EF conditions or in a significant association between a more externalized attentional focus and better performance (Lohse et al., 2014a; Becker and Fairbrother, 2019; Kakvandi et al., 2025). Four studies reported conditional or mixed findings, with EF-favoring effects observed only at a marginal level of significance, in directional analyses, during specific stages of practice or sessions, or within particular participant groups (Hitchcock and Sherwood, 2018; Lohse et al., 2010; Marchant et al., 2009; Emanuel et al., 2008). The remaining three studies found no significant EF–IF differences (Ghorbani et al., 2019; Querfurth et al., 2016; Simpson et al., 2022). Taken together, the acquisition-phase evidence did not demonstrate a consistent advantage of either attentional focus strategy, although EF-favoring effects were observed in several studies or specific experimental conditions (Table 3).

**Table 3:** Direct comparison of EF and IF in the acquisition phase.

Finding category	Study	Outcome measure	Main finding
Significant EF-favoring findings	Lohse et al., 2014a	Absolute error	FOA effect sig; linear contrast sig (more external < more internal)
	Becker and Fairbrother, 2019	Error scores	EF < IF; group × block sig
	Kakvandi et al., 2025	Mean radial error	EF < IF; focus × block sig; WMC NS.
Conditional or mixed findings	Hitchcock and Sherwood, 2018	Mean radial error	EF < IF (*p* =0.050)
	Lohse et al., 2010	Absolute error	EF < IF (directional test)
	Marchant et al., 2009	Error scores	Session 1: IF vs. EF NS; Session 2: EF < IF; bull's-eye hits: EF > IF
	Emanuel et al., 2008	Mean radial error	Adults improved significantly across blocks under EF; Children showed no clear pattern.
No significant EF–IF difference	Ghorbani et al., 2019	Scores	IF vs. EF NS; block effect sig; group × block NS
	Querfurth et al., 2016	Mean radial error	IF vs. EF NS
	Simpson et al., 2022	Mean radial error	IF vs. EF NS

EF, external focus; IF, internal focus; FOA, focus of attention; WMC, working memory capacity; sig, statistically significant; NS, not significant.

“ × ” indicates an interaction effect. For error-based measures, lower values indicate better performance; for score-based measures and bull's-eye hits, higher values indicate better performance.

#### 3.4.2 Retention phase

A total of five studies reported direct comparisons during the retention phase. Three studies reported an EF advantage at retention intervals of 1 day or 72 h (Ghorbani et al., 2019; Becker and Fairbrother, 2019; Kakvandi et al., 2025), whereas two studies found no significant EF–IF differences (Emanuel et al., 2008; Simpson et al., 2022). In Emanuel et al. (2008), adults outperformed children overall, but no significant age × attentional focus interaction was observed; Simpson et al. (2022) likewise reported no significant EF–IF difference at the 10-day retention test. Taken together, some retention findings favored EF, but the small number of studies and variation in retention intervals limit conclusions regarding a consistent retention advantage (Table 4).

**Table 4:** Direct comparison of EF and IF in the retention phase.

Finding category	Study	Outcome measure	Retention interval	Main finding
Significant EF-favoring findings	Ghorbani et al., 2019	Scores	1 day	EF > IF; IF vs. CON NS
	Becker and Fairbrother, 2019	Error scores	1 day	EF < IF
	Kakvandi et al., 2025	Mean radial error	72 h	EF < IF; focus × time sig; only EF improved from pretest to retention; WMC NS.
No significant EF–IF difference	Emanuel et al., 2008	Mean radial error	1 day	Age × EF/IF NS; adults > children overall.
	Simpson et al., 2022	Mean radial error	10 days	IF vs. EF NS

EF, external focus; IF, internal focus; CON, control; WMC, working memory capacity; NS, not significant; sig, statistically significant.

“ × ” indicates an interaction effect. For error-based measures, lower values indicate better performance; for score-based measures, higher values indicate better performance.

#### 3.4.3 Transfer phase

A total of three studies reported direct comparisons during the transfer phase, making it the phase with the least available evidence. Two studies found no significant EF–IF differences in transfer tasks involving non-dominant-hand throwing or increased throwing distance (Ghorbani et al., 2019; Simpson et al., 2022). The remaining study showed an age-dependent pattern: adults performed better under EF than IF, whereas children showed the opposite pattern during transfer (Emanuel et al., 2008). Taken together, transfer evidence was sparse and did not show a consistent EF–IF pattern (Table 5).

**Table 5:** Direct comparison of EF and IF in the transfer phase.

Finding category	Study	Outcome measure	Transfer condition	Main finding
Conditional or mixed findings	Emanuel et al., 2008	Mean radial error	Increased throwing distance	Adults: EF < IF; Children: IF < EF
No significant EF–IF difference	Simpson et al., 2022	Mean radial error	Increased throwing distance	IF vs. EF NS
	Ghorbani et al., 2019	Scores	Transfer with the left (non-dominant) hand	IF vs. EF NS

EF, external focus; IF, internal focus; NS, not significant. For error-based measures, lower values indicate better performance; for score-based measures, higher values indicate better performance.

### 3.5 Potential sources of variation in EF–IF findings

Beyond direct comparisons, several studies examined whether EF–IF findings varied according to task demands, experimental context, and individual characteristics. Based on their primary focus, these findings were synthesized into two domains: task- and context-related factors and individual-related factors.

#### 3.5.1 Task- and context-related factors

Task- and context-related factors mainly involved task demands, instructional format, and practice and test arrangements. Overall, the available evidence did not support a stable EF advantage, and findings varied across experimental settings (Table 6).

**Table 6:** Effects of task- and context-related factors on EF–IF differences.

Factor domain	Studies	Specific factor	Study context	Outcome measure	Results
Task demands	Asadi et al., 2022	Cognitive load	Low vs. high cognitive load × EF/IF	Mean radial error	AQ: Low load > high load; EF favored descriptively; focus main effect approached significance.
	Sherwood et al., 2020, Exp. 1–2	Dual-task demands	Dart throwing with/without tone-estimation task	Mean radial error	Exp. 1: EF+EST < IF+EST, but overall EF/IF NS. Exp. 2: EF < IF; EST < No-EST; order-related interactions were significant.
	Sherwood et al., 2020, Exp. 3	Task goal	Dart-goal task vs. force-goal task	Mean radial error	AQ: EFD < IFD, whereas EFF vs. IFF was NS.
	Sherwood et al., 2020, Exp. 4	Force output level	20% MF vs. 50% MF under dart-goal and force-goal conditions	Mean radial error	AQ: EF < IF; 20% MF < 50% MF; force-goal × task interaction was significant.
Instructional format	Neugebauer et al., 2020	Instructional format/perceptual channel	Visual vs. kinesthetic instruction × EF/IF	Radial distance	AQ: EF vs. IF NS; VIS vs. KIN NS.
	Sampson et al., 2025	Self-talk format	External instructional vs. internal instructional vs. motivational self-talk	Mean radial error	AQ: EIST < IIST; MST < IIST; EIST vs. MST NS.
Practice/testing arrangement	Marchant et al., 2009	Condition order/instruction preference	IF → EF vs. EF → IF; instruction preference	Error scores	Session 1: IF vs. EF NS; IF → EF improved, EF → IF worsened. Session 2: EF < IF; EF-preference group performed better under EF.
	Lohse et al., 2014b, Exp. 1	Testing focus	Acquisition focus × testing focus; four FOA combinations (EE, EI, IE, II); short acquisition; wrist-weight transfer	Mean radial error	AQ: acquisition focus NS. RT/TT: testing focus effect (EE + IE < EI + II).
	Lohse et al., 2014b, Exp. 2	Testing focus; practice amount	Acquisition focus × testing focus; four FOA combinations (EE, EI, IE, II); longer acquisition; wrist-weight transfer	Mean radial error	AQ: acquisition focus NS. RT: testing focus effect (EE + IE < EI + II). TT: no main effect of acquisition focus or testing focus.
	Shafizadeh et al., 2013	Practice type	EF/IF during demonstration × physical/observational practice	Scores	AQ: EF vs. IF NS; practice type not examined. RT/TT: EF > IF after controlling self-efficacy; physical vs. observational NS.

EF, external focus; IF, internal focus; AQ, acquisition phase; RT, retention test; TT, transfer test; NS, not significant; EST, tone-estimation task; No-EST, no tone-estimation task; EFD, external focus in the dart-goal task; IFD, internal focus in the dart-goal task; EFF, external focus in the force-goal task; IFF, internal focus in the force-goal task; MF, maximal force; VIS, visual instruction; KIN, kinesthetic instruction; EIST, external instructional self-talk; IIST, internal instructional self-talk; MST, motivational self-talk; FOA, focus of attention; EE, external acquisition–external test; EI, external acquisition–internal test; IE, internal acquisition–external test; II, internal acquisition–internal test.

“ × ” indicates an interaction or combination of factors. For error-based measures, lower values indicate better performance; for score-based measures, higher values indicate better performance.

Regarding task demands, factors such as cognitive load, dual-task requirements, task goals, and force output levels were associated with differences in the pattern of results. The findings suggest that EF–IF differences may depend on additional task demands, goal structure, and movement requirements. Under some conditions, performance tended to favor EF, whereas under others no significant difference was observed (Asadi et al., 2022; Sherwood et al., 2020).

Regarding instructional format, different types of cues were associated with different outcomes. Comparisons between visually oriented and kinesthetically oriented instructions did not reveal a stable EF–IF difference (Neugebauer et al., 2020). Under self-talk conditions, both externally instructional self-talk and motivational self-talk were superior to internally instructional self-talk (Sampson et al., 2025).

Regarding practice and test arrangements, the relevant studies examined factors including condition order, instructional preference, the focus adopted at test, and practice format or amount of practice. Two studies did not observe a clear EF–IF difference during the acquisition phase. Subsequent testing showed that, in one study, performance was better when EF was adopted during the test phase than when IF was adopted, regardless of the attentional focus used during acquisition (Lohse et al., 2014b). In the other study, EF was superior to IF in both the retention and transfer phases after controlling for self-efficacy (Shafizadeh et al., 2013). In addition, condition order and instructional preference were also related to the observed pattern of results: performance improved when the focus shifted from IF to EF and declined when it shifted from EF to IF, and participants who preferred EF instructions performed better under EF conditions (Marchant et al., 2009).

Overall, these findings indicate that task- and context-related variables are associated with how EF–IF differences emerge, although the specific patterns vary across studies.

#### 3.5.2 Individual-related factors

Evidence on individual-related factors mainly concerned propensity for conscious control, related cognitive control characteristics, skill level, age, and visuospatial working memory capacity (Table 7).

**Table 7:** Effects of individual-related factors on EF–IF differences.

Factor domain	Studies	Specific factor	Study context	Outcome measure	Results
Conscious control-related traits	Tse and van Ginneken, 2017	Conscious control propensity	High vs. low conscious control propensity × EF/IF	Mean radial error	AQ: no instruction effect. RT/TT: matching effects emerged (high-CCP children performed better under IF; low-CCP children performed better under EF).
	van Ginneken et al., 2017	Conscious control/CMP	Conscious monitoring vs. conscious control; high vs. low CMP	Mean radial error	AQ: focus main effect NS; effects varied by CMP and order under conscious-control conditions.
Skill/developmental characteristics	Schorer et al., 2012	Skill level	Experts vs. novices	Mean radial error	AQ: Experts outperformed novices across conditions; no stable EF advantage within either group.
	Emanuel et al., 2008	Age	Children vs. adults × EF/IF	Mean radial error	AQ: age-related effects; adults favored EF, whereas children showed no stable EF/IF difference. RT: age × focus NS; adults outperformed children overall. TT: adults EF < IF; children IF < EF.
Cognitive capacity	Kakvandi et al., 2025	Visuospatial WMC	High vs. low visuospatial WMC × EF/IF	Mean radial error	AQ/RT: EF < IF; WMC NS. TT (longer distance): EF < IF; low WMC < high WMC. TT (dual-task): EF < IF; WMC NS.

AQ, acquisition phase; RT, retention test; TT, transfer test; EF, external focus; IF, internal focus; CCP, conscious control propensity; CMP, conscious motor processing; WMC, working memory capacity; NS, not significant. “ × ” indicates an interaction or combination of factors. For mean radial error, lower values indicate better performance.

Regarding propensity for conscious control and related cognitive-control characteristics, the available studies did not show a clear main effect of attentional focus during acquisition. However, findings from retention and transfer suggest that responses to EF and IF may depend on an individual's propensity for conscious control (Tse and van Ginneken, 2017). Relatedly, EF–IF differences during acquisition appeared to vary with conscious motor processing and condition order (van Ginneken et al., 2017).

Regarding skill level and age, the available evidence suggests that participant background may influence the pattern of findings, although no stable or uniform pattern has been established. Experts generally outperformed novices overall, but no consistent EF advantage was observed within either group (Schorer et al., 2012). In age-related research, adults appeared more likely to show findings favoring EF at certain stages, whereas children did not show the same pattern (Emanuel et al., 2008).

Regarding visuospatial working memory capacity, lower radial error under EF than IF was observed across acquisition, retention, and both transfer tasks, whereas WMC-related differences emerged only in the longer-distance transfer task; no significant WMC effect was found during acquisition, retention, or dual-task transfer (Kakvandi et al., 2025).

Overall, evidence for individual-related variation remains preliminary, as most characteristics were examined in only one or two studies. Although EF–IF differences appeared to vary according to some individual characteristics, the available evidence is insufficient to establish a consistent individual-specific pattern.

## 4 Discussion

This review systematically synthesized the effects of IF and EF on dart-throwing performance across 19 studies comprising 23 experimental units. Overall, the evidence showed a mixed and context-dependent pattern rather than a consistent advantage of either attentional focus strategy. During acquisition, statistically significant EF-favoring findings were reported in three studies, whereas the remaining studies showed conditional, mixed, or non-significant findings. Retention evidence was limited, with three of five studies favoring EF, while transfer evidence was sparse and showed no consistent EF–IF pattern. Taken together, EF-favoring effects were observed under some conditions, but the current evidence is insufficient to support a general advantage of EF over IF across phases and contexts.

The relatively equivocal pattern observed in dart throwing differs from the generally more favorable effects of EF reported in broader cross-task reviews and meta-analyses (Chua et al., 2021; Kim et al., 2017; Hawkins et al., 2025; Wulf, 2013). Several explanations may account for this discrepancy. Dart throwing may impose task-specific perceptual and motor demands that constrain the effects of attentional focus, while substantial methodological variation across the included studies may also contribute to differences in findings. In addition, evidence for retention and transfer remains limited, making it difficult to determine whether effects observed during practice persist as motor learning. Concerns regarding reporting bias and between-study heterogeneity in the broader attentional focus literature further warrant caution when interpreting apparent EF advantages (McKay et al., 2024). Recent boundary evidence likewise suggests that IF may be advantageous under specific learner and task conditions (Becker et al., 2025). Thus, the present findings do not necessarily contradict the broader literature, but indicate that its general conclusions may not translate uniformly to dart throwing.

Methodological heterogeneity also warrants consideration when interpreting the present findings. The included studies varied in design, participant characteristics, outcome measures, assessment phases, attentional focus instructions, and practice protocols. Although instruction content and practice dosage differed considerably, neither showed a consistent descriptive relationship with the direction of EF–IF findings. Thus, the mixed results cannot readily be attributed to a single methodological characteristic, while the broader variation across studies limits direct comparability and confidence in any general conclusion regarding EF superiority. Similar methodological concerns have also been noted in related motor-learning research (Bacelar et al., 2024).

The distinction between immediate performance and motor learning remains important when interpreting phase-specific findings. Performance during acquisition reflects behavior under ongoing practice conditions and should not be equated directly with relatively durable learning, for which delayed retention and transfer tests provide more informative evidence (Kantak and Winstein, 2012). In the present review, several studies reported EF-favoring findings during acquisition, and some retention studies also favored EF; however, the limited number of retention studies and the sparse and inconsistent transfer evidence preclude firm conclusions about whether EF-favoring effects observed during practice persist over time or generalize to altered task conditions. Practice–test specificity may further contribute to variation in subsequent performance (Shea and Kohl, 1990). Accordingly, the current evidence is insufficient to determine whether the observed EF-favoring effects primarily reflect immediate performance benefits or extend to durable motor learning in dart throwing.

Task- and context-related factors may contribute to the variation in EF–IF findings across studies. The included studies suggest that attentional focus effects may differ according to cognitive load, dual-task requirements, task goals, force output demands, instructional format, and the arrangement of practice and testing. These findings indicate that EF–IF differences in dart throwing are likely to depend partly on the specific task and experimental context rather than operating independently of them. Given its self-paced, visually guided, and accuracy-oriented nature, dart throwing may also be particularly sensitive to variations in cognitive demands and testing conditions. Theoretically, such variation can be considered in relation to the common-coding/ideomotor perspective, the constrained action hypothesis, and OPTIMAL theory, which propose that attention directed toward movement effects may facilitate efficient movement organization and reduce explicit movement monitoring (Wulf and Prinz, 2001; Wulf et al., 2001; Wulf and Lewthwaite, 2016). Related evidence further suggests that EF may promote more automatic modes of motor control (Kal et al., 2013), while research on dual-task demands, attentional resources, and self-talk indicates that additional processing demands and instructional format may alter how attentional cues influence performance (Johnston and Pashler, 1998; Theodorakis et al., 2000; Hatzigeorgiadis and Galanis, 2017). However, these theoretical accounts should be regarded as interpretive frameworks rather than direct evidence that the mechanisms underlying the mixed EF–IF findings in dart throwing have been established.

Evidence concerning individual-level sources of variation remains preliminary, as most candidate characteristics were examined in only one or two studies. Some findings suggest that propensity for conscious control may be associated with differential responses to EF and IF, with reinvestment theory providing a plausible interpretive framework for such variation (Masters and Maxwell, 2008). Differences related to age, skill level, and visuospatial working memory capacity have also been reported, but the available evidence is too limited to establish these characteristics as reliable moderators of attentional focus effects. Although developmental stage and motor–cognitive characteristics may plausibly influence responses to attentional instructions (Musculus and Raab, 2022), further evidence is needed before consistent individual-specific recommendations can be made.

Overall, the principal contribution of this review lies in showing that EF–IF effects in dart throwing are less consistent than a general EF-superiority account would imply. Although EF-favoring findings were observed in several studies, their occurrence varied across test phases and experimental contexts, while substantial methodological heterogeneity limits confidence in any overarching conclusion regarding EF superiority. From a practical perspective, EF may therefore be considered a potentially useful instructional option in dart-throwing practice, but the current evidence does not support routinely prioritizing EF over IF across learners and practice contexts. Attentional instructions should instead be selected with consideration of task demands, practice goals, and learner characteristics, rather than on the assumption that one focus is universally optimal. Further task-specific research using more standardized methods and delayed retention and transfer assessments is needed to determine under what conditions and for whom particular attentional focus strategies are most effective.

## 5 Limitations and future directions

Several limitations should be considered when interpreting the present findings. First, the included studies employed diverse experimental designs, including within-subject, between-subject, and mixed designs, together with differences in outcome reporting and assessment phases. These variations complicated direct comparison and quantitative synthesis, particularly because some studies did not provide sufficient information for deriving comparable effect-size estimates. Accordingly, the present review relied primarily on narrative synthesis. Second, the evidence base was concentrated largely on the acquisition phase, whereas relatively few studies examined delayed retention and transfer, limiting conclusions regarding the durability and generalizability of EF–IF effects. Third, most participants were healthy young novices, with comparatively limited evidence from children, skilled performers, and other populations. Fourth, the MMAT appraisal identified recurring limitations in the reporting of randomization and blinding, together with uncertainties regarding intervention implementation or outcome data in some studies, which should be considered when interpreting the robustness of the findings. Finally, because the review focused on explicit IF and EF instructional manipulations, the findings may not generalize to indirect or implicit methods of directing attentional focus.

Future research should prioritize greater methodological standardization, particularly in the reporting of attentional focus instructions, practice dosage, outcome measures, and assessment procedures, to improve comparability across studies. Greater emphasis should also be placed on delayed retention and transfer tests to determine whether EF-favoring effects observed during practice persist over time and generalize to altered task conditions. Studies involving broader ranges of age, skill level, and training background are needed to examine whether EF–IF effects vary systematically across learner populations. Finally, adequately powered and methodologically rigorous studies should test task-, context-, and learner-related sources of variation more systematically, helping to establish under what conditions and for whom particular attentional focus strategies are most effective.

## 6 Conclusion

This systematic review indicates that EF–IF effects in dart throwing are mixed and context dependent rather than consistently favoring one attentional focus strategy. Although several studies reported EF-favoring findings during acquisition and retention, other studies reported conditional, mixed, or non-significant effects, and transfer evidence remains sparse and inconsistent. Substantial methodological heterogeneity and the limited evidence from delayed retention and transfer assessments further constrain confidence in any general EF advantage. Accordingly, EF may represent a potentially useful instructional option in dart throwing, but the current evidence does not support its routine prioritization over IF. More standardized and task-specific research is needed to determine under what conditions and for whom different attentional focus strategies are most effective.

## Data Availability

The original contributions presented in the study are included in the article/Supplementary material, further inquiries can be directed to the corresponding author.
