# Supplementary material for: Effects of internal and external attentional focus on dart-throwing performance: a systematic review
Source: Front Psychol. 2026 Sep 3;17:1923857. doi: 10.3389/fpsyg.2026.1923857 (PMC13581625; doi:10.3389/fpsyg.2026.1923857)
Supplement: Supplementary file 1 [file Table_1.docx]

Supplementary Material

# 1 Supplementary Tables

Supplementary Table 1. Database-specific search strategies.

| Web of Science | TS=((("darts" OR "dart" OR "dart throwing" OR "dart throw*" OR "dart-throw*" OR "target throwing" OR "goal-directed throwing" OR "aiming task*" OR "target task*") OR (("target*" OR "aim*" OR "goal-directed" OR "goal directed") NEAR/3 throw*)) AND ("attentional focus" OR "focus of attention" OR "attention focus" OR "external focus" OR "internal focus" OR "attentional instruction*" OR "focus instruction*" OR "instructional focus" OR "movement effect" OR "constrained action" OR "constrained action hypothesis" OR "focus distance" OR "distal focus" OR "proximal focus")) |
| --- | --- |
| Scopus | TITLE-ABS-KEY(((darts OR dart OR "dart throwing" OR "dart throw*" OR "dart-throw*" OR "target throwing" OR "goal-directed throwing" OR "aiming task*" OR "target task*") OR (("target*" OR "aim*" OR "goal-directed" OR "goal directed") W/3 throw*)) AND ("attentional focus" OR "focus of attention" OR "attention focus" OR "external focus" OR "internal focus" OR "attentional instruction*" OR "focus instruction*" OR "instructional focus" OR "movement effect*" OR "constrained action" OR "constrained action hypothesis" OR "focus distance" OR "distal focus" OR "proximal focus")) |
| PubMed | ((("darts"[tiab] OR "dart"[tiab] OR "dart throwing"[tiab] OR "dart throw*"[tiab] OR "dart-throw*"[tiab] OR "target throwing"[tiab] OR "goal-directed throwing"[tiab] OR "aiming task*"[tiab] OR "target task*"[tiab]) OR ((target*[tiab] OR aim*[tiab] OR goal-directed[tiab] OR "goal directed"[tiab]) AND throw*[tiab])) AND ("attentional focus"[tiab] OR "focus of attention"[tiab] OR "attention focus"[tiab] OR "external focus"[tiab] OR "internal focus"[tiab] OR "attentional instruction*"[tiab] OR "focus instruction*"[tiab] OR "instructional focus"[tiab] OR "movement effect*"[tiab] OR "constrained action"[tiab] OR "constrained action hypothesis"[tiab] OR "focus distance"[tiab] OR "distal focus"[tiab] OR "proximal focus"[tiab])) |
| Embase | ((('darts':ti,ab,kw OR 'dart':ti,ab,kw OR 'dart throwing':ti,ab,kw OR 'dart throw*':ti,ab,kw OR 'dart-throw*':ti,ab,kw OR 'target throwing':ti,ab,kw OR 'goal-directed throwing':ti,ab,kw OR 'aiming task*':ti,ab,kw OR 'target task*':ti,ab,kw) OR ((target* OR aim* OR 'goal-directed' OR 'goal directed') NEAR/3 throw*):ti,ab,kw) AND ('attentional focus':ti,ab,kw OR 'focus of attention':ti,ab,kw OR 'attention focus':ti,ab,kw OR 'external focus':ti,ab,kw OR 'internal focus':ti,ab,kw OR 'attentional instruction*':ti,ab,kw OR 'focus instruction*':ti,ab,kw OR 'instructional focus':ti,ab,kw OR 'movement effect*':ti,ab,kw OR 'constrained action':ti,ab,kw OR 'constrained action hypothesis':ti,ab,kw OR 'focus distance':ti,ab,kw OR 'distal focus':ti,ab,kw OR 'proximal focus':ti,ab,kw)) |

Supplementary Table 2. Instructional and practice characteristics of the included studies and experimental units.

| **Study** | **IF instruction** | **EF instruction** | **Instruction mode** | **Practice dosage** | **Manipulation check** |
| --- | --- | --- | --- | --- | --- |
| Asadi et al. (2022) | Mentally focus on the motion of the throwing arm | Mentally focus on the flight of the dart | Oral + written | 3 throws × 6 blocks | Yes |
| Lohse et al. (2014a) | Focus on the motion of your arm | Release: Focus on the dart leaving your hand; Trajectory: Focus on the flight of the dart;  Board: Focus on the bull’s-eye | Oral | 3 throws × 5 blocks × 4 sessions | NR |
| Ghorbani et al. (2019) | Focus on the hand while throwing the dart | Focus on the bullseye while throwing the dart | Oral | 6 throws × 10 blocks | Yes |
| Hitchcock & Sherwood (2018) | Focus on the elbow angle at dart release | Focus on the initial flight of the dart | Oral | 3 throws × 8 blocks | NR |
| Becker & Fairbrother (2019) | “Accelerate the forearm”; “Straight arm path”; “Hand to target” | Accelerate the dart; Straight dart path; Hit the target | Self-talk | 5 throws × 12 blocks | Yes |
| Lohse et al. (2010) | Focus on the movement of your arm and think about how you are moving | Focus on the flight of the dart and think about how it should fly | Oral | 3 throws × 7 blocks | NR |
| Neugebauer et al. (2020) | Visual: “Concentrate on your eye”;  Kinesthetic: “Concentrate on your hand” | Visual: Concentrate on the bullseye;  Kinesthetic: Concentrate on the dart | NR | 50 throws × 3 sessions | NR |
| Querfurth et al. (2016) | Mentally focus on the movement of your arm | Mentally focus on the flight of the dart | NR | 3 throws × 3 sets × 2 blocks | No |
| Sampson et al. (2025) | “Extend the elbow”; “Flick the wrist”; “Bend the elbow”; “Smooth elbow” | Aim for the bullseye; Aim for the target; Hit the bullseye; Hit the target | Self-talk | 5 throws × 3 sets | Yes |
| Schorer et al. (2012) | Concentrate on the return point of your movement | Concentrate on the bull’s eye | Oral | 3 throws × 4 series | NR |
| Sherwood et al. (2020), Exp. 1 | Mentally focus on the motion of the arm | Mentally focus on the flight of the dart | NR | 24 throws × 2 task conditions | Yes |
| Sherwood et al. (2020), Exp. 2 | Mentally focus on the motion of the arm | Mentally focus on the flight of the dart | NR | 24 throws × 4 conditions | Yes |
| Sherwood et al. (2020), Exp. 3 | Dart: Focus on elbow movement and estimate elbow angle at release;  Force: Focus on forearm muscles and judge muscle activity | Dart: Focus on dart flight and estimate its trajectory;  Force: Focus on dynamometer-pointer movement and judge the distance moved | NR | 15 throws × 4 conditions | NR |
| Sherwood et al. (2020), Exp. 4 | Dart: Focus on elbow movement and estimate elbow angle at release;  Force: Focus on forearm muscles and judge muscle activity | Dart: Focus on dart flight and estimate its trajectory;  Force: Focus on dynamometer-pointer movement and judge the distance moved | NR | 15 throws × 4 conditions | NR |
| Simpson et al. (2022) | Focus on the feeling of the arm bending, muscle contraction, and full arm extension | Visually focus on the bullseye and concentrate on the flight and motion of the dart | NR | 3 throws × 6 blocks × 2 acquisition phases | Yes |
| Tse & van Ginneken (2017) | Focus on finger motion, grasp, and elbow flexion–extension | Focus on the target and the dart’s flight, weight, and position | Oral | 10 throws × 5 blocks | Yes |
| van Ginneken et al. (2017) | Monitoring: Focus on the motion of your arm and be aware of how it moves; Control: Focus on the motion of your arm and think about how it should move | Monitoring: Focus on the motion of the dart and be aware of how it flies;  Control: Focus on the motion of the dart and think about how it should fly | Oral | 45 throws/condition | Yes |
| Marchant et al. (2009) | Focus on the movement of the arm during the draw-back and throw, and on dart release | Focus on the center of the dartboard and throw when focused | Oral + written | Session 1: 4 throws × 5 blocks/condition;  Session 2: 4 throws × 10 blocks | Yes |
| Emanuel et al. (2008) | Focus on arm and hand movements (elbow, wrist, and fingers) | Focus on the dart, its flight, and the target | Oral | 10 throws × 5 blocks | Yes |
| Kakvandi et al. (2025) | Focus on your fingers and elbow extension | Focus on the dart and throwing path | Oral | 10 throws × 5 blocks | No |
| Lohse et al. (2014b), Exp. 1 | Focus on the motion of your arm | Focus on the flight of the dart | Oral | 3 throws × 10 blocks | NR |
| Lohse et al. (2014b), Exp. 2 | Focus on the motion of your arm | Focus on the flight of the dart | Oral | 9 throws × 10 blocks × 2 days + 9 throws × 2 blocks | Yes |
| Shafizadeh et al. (2013) | Focus on limb movements (arm/elbow and weight transfer) | Focus on the dart, target, and movement effects | Oral | 6 throws × 10 blocks | Yes |

Note. IF = internal focus; EF = external focus; NR = not reported. Practice dosage reflects the trial structure reported in the original study. Manipulation check: Yes = explicitly assessed; No = explicitly not conducted; NR = not reported.
